# Clinical observations from a study comparing two different treatments for bluetongue in sheep

**DOI:** 10.1186/s13620-026-00351-5

**Published:** 2026-06-22

**Authors:** Ruurd Jorritsma, Hannes J. C. Bijkerk, Reynaldo Martina, Inge M. van Geijlswijk

**Affiliations:** 1 Faculty of Veterinary Medicine, Population Health Sciences, Sustainable Ruminant Health, Utrecht University, Yalelaan 7, Utrecht, 3584 CL the Netherlands; 2 Faculty of Veterinary Medicine, Population Health Sciences, Utrecht University, Yalelaan 7, Utrecht, 3584 CL the Netherlands; 3 Faculty of Veterinary Medicine, Department of Population Health Sciences, Division of Institute for Risk Assessment Sciences (IRAS), Pharmacy, Utrecht University, Yalelaan 106, Utrecht, 3584 CM the Netherlands

**Keywords:** Bluetongue, Treatment, Sheep, Paracetamol, Antihistamine

## Abstract

In September 2023, the official national reference laboratory confirmed the presence of BTV serotype 3 in The Netherlands. In the following weeks, it appeared that the infection caused very severe clinical signs in sheep, but it was challenging for the involved veterinary clinicians to select appropriate labeled drugs to treat them. The objective of this study was therefore to document the clinical presentation and survival outcomes of sheep affected by bluetongue and to evaluate the effects of a treatment protocol with and without antihistamines.

From a group of 83 sheep with acute and clear clinical signs of bluetongue, one group of 41 sheep received a single injection of meloxicam (1 mg/kg) followed by oral paracetamol tablets (10–20 mg/kg, 3dd for 3d). Another group with 42 sheep received the same treatment with the addition of an antihistamine injection (chlorphenamine maleate, 1 mg/kg, 1dd for 3d). Clinical signs and survival rates were monitored for all sheep. Logistic regression and survival analyses were performed to evaluate the data.

An overall case fatality rate of 57% was observed, which illustrates the challenge in treating infected naïve animals with BTV-3. The most commonly encountered clinical signs were excessive salivation, dyspnea, depression, and lameness. Severe salivation was associated with significantly reduced survival time. The mean survival time for the group treated with antihistamines was 0.8 days, whereas it was 2.5 days for the group without antihistamines.

The results of this study may not be representative of bluetongue infections caused by other serotypes or under different conditions. Based on our findings, however, we cannot recommend the use of antihistamines for treating bluetongue-affected sheep, as they did not improve survival. We would like to emphasize the importance of timely decision-making regarding the continuation or cessation of treatment, given the typically low survival rates in clinically affected sheep.

## Background

Bluetongue virus (BTV) is an orbivirus that affects sheep in particular, while goats and cattle are also susceptible. Of the many serotypes, BTV-1 to BTV-24 are transmitted by *Culicoides* spp and often cause high morbidity of infected animals [16]. The infections with these serotypes result in injuries of endothelial cells of capillaries in particular, leading to a range of clinical signs including for example oral erosions and ulcers, hyperemia and oedema of lips, cyanosis of the tongue, coronitis, stillbirths and abortions [16]. Other serotypes are considered atypical as they are non-pathogenic and not transmitted by vectors [16]. Within the EU, BTV infections are notifiable and therefore monitored based on regulations EU 216/429 and EU 2018/88.

Up to 1998, BTV infections usually occurred in tropical and subtropical parts of the world, as this was the habitat of the competent vector. It is hypothesized that changes in climate, terrestrial habitat conditions, wind direction and higher livestock densities are responsible for increased virus persistence during winter, the northward expansion of the vector and the transmission of indigenous European *Culicoides* species, resulting in the introduction and spread of BTV into Europe [1, 8, 15]. Among these was the BTV-8 outbreak in The Netherlands. The country was declared free again in February 2012.

Many preventive and control strategies for BTV infections have an impact on the presence of or the contact between susceptible animals and the vector. These include husbandry measures, destruction of breeding sites of the vector, and the use of insecticides and repellents. In addition, there are several vaccines licensed for commercial use, which are used during outbreaks and in endemic situations [11]. Once infected prior recommendations included treating the animals with antipyretic, antihistamine, or non-steroidal anti-inflammatory (NSAID) drugs and provision with soft food and water [16].

In September 2023, the official national reference laboratory confirmed a new incursion of BTV into The Netherlands. The virus was soon thereafter classified as serotype 3. As all animals in the country were naïve for the infection, the virus spread rapidly in the following months, resulting in a large-scale epidemic that affected thousands of ruminants across the country [7]. On September 19th, the first clinical signs of bluetongue were observed in 3 rams at the university teaching farm, which tested all positive for BTV-3 via RT-PCR. At the same time, a large proportion of the adjacent dairy herd also tested positive [9]. Given the oral reports from local veterinarians, we expected that the infection would soon result in severe clinical signs in a large proportion of the sheep in the herd that needed long-term treatment and support.

While there is currently a study available on the outcome of hospitalized sheep with bluetongue virus disease, there are no quantitative studies on treatment effectivities in clinical practice in an epidemic situation [5]. Practicing veterinarians therefore often used treatment guidelines based on clinical and pharmaceutical expertise during the Dutch epidemic of 2023 [10]. It was also clear that that long-term treatments with NSAIDs would deteriorate the health condition of sheep in this outbreak as it was shown that many BTV-3 infected sheep became dehydrated and already had laminitis and papillary necrosis of the kidneys [2]. We considered the use of corticosteroids, but as many ewes were several months pregnant and injections with corticoids may result in abortion in this species, systematic treatment would likely be detrimental. As a careful pregnancy check, followed by a tailored treatment, was not feasible during the outbreak situation, we concluded that corticoid treatments were in this case not a good option.

While paracetamol was not licensed for sheep, we concluded that paracetamol could be used in sheep legally under the EU cascade regulation (EU 2019/6), as a Maximum Residue Limit (MRL) was established for swine (EU 37/2010). Given other studies, sheep are believed to metabolize paracetamol effectively [4, 12]. Moreover, paracetamol was used for postsurgical pain relief in sheep at 10 mg/kg intravenously and 15 mg/kg orally every 12 h [13]. Considering additional pharmacokinetic data from goats, we concluded that a dosage of 10–20 mg/kg orally, three times daily, would be appropriate.

In addition, we considered the use of an antihistamine for its potential anti-inflammatory effects in BTV infections, as was previously mentioned by Saminathan et al. [16]. There are indeed indications that antihistamines are not only effective in the mediation of allergic reactions, but may also have the potential to act as anti-infectives [17]. We found anecdotal evidence for this effect in cattle with a BRSV infection, while the clinical evidence of this effect seems lacking in humans [6, 17]. Chlorphenamine is an antihistamine drug that has an MRL opinion for all food-producing species, which allows the use of chlorphenamine for sheep in the EU.

Given these considerations, we decided to document the effect of a treatment protocol that is allowed according to EU legislation, consisting of single NSAID injections combined with paracetamol tablets on clinical signs and survival of bluetongue-affected sheep and to evaluate the additive effect of antihistamines.

## Materials and methods

### Animals and treatment

This study was conducted in a herd at a teaching farm with 260 sheep, including ewes of varying age and their lambs of around 9 months of age. After the confirmation of the presence of BTV-3 virus in many animals of the neighboring dairy farm and in three rams from the same flock, we included sheep with clear clinical signs of bluetongue in the study without further laboratory confirmation between October and November 2023. Given the official free-status of The Netherlands, all sheep were naïve for BTV-3 before this outbreak.

All sheep were treated at diagnosis with a single subcutaneous injection of meloxicam (1 mg/kg; Novem® 20 mg/mL, Boehringer Ingelheim) on day 0. From day 1 onward, they were all administered 10–20 mg/kg of oral paracetamol (500 mg tablets; Centrafarm) three times daily by animal caretakers. Moreover, one group (AH) received in addition also antihistamines, whereas the other group (NAH) did not. The antihistamine treatment of the AH group consisted of chlorphenamine maleate (1 mg/kg; Ancesol® 10 mg/mL, AST Pharma) via injection once daily on days 1, 2, and 3. Each newly diagnosed case was alternately assigned to either treatment group.

Assuming a two-sided significance level of 5% and a power of 80%, we estimated that 66 affected sheep were needed to detect that the time until recovery of the no antihistamine (NAH) group was twice as long as for the antihistamine (AH) group. All included sheep were individually housed within the same shed. Moreover, we provided all affected sheep with soft food, minimized stress, florfenicol was administered as clinically indicated for secondary infections of the respiratory tract. Oral rehydration was offered to the animals as needed.

The following clinical signs were scored daily: abnormal posture, swollen head, decreased feed intake, increased respiratory rate, lesions on the nose, lameness, nasal discharge, depression, excessive salivation, teat lesions, and dyspnea. Dyspnea was scored as present or absent, while all others were scored as 0 (not observed), 1 (mild), 2 (moderate), or 3 (severe). Scoring was performed by trained veterinary students who were blinded to treatment allocation. In addition, the rectal temperature was recorded. Parenteral treatments with meloxicam and chlorphenamine were administered by veterinarians who were blinded to the clinical scoring.

The veterinarian euthanized animals whose overall clinical condition worsened in the opinion of the animal caretaker (blinded to both treatment and clinical scoring) compared to the previous day using pentobarbital (Euthanimal® 40 mg/mL, Alfasan).

### Statistical analysis

Clinical scores were summarized by treatment group (AH vs. NAH) for the baseline and post-baseline periods. Baseline scores were defined as the most severe score recorded on days 0 and 1. Post-baseline measurements were taken from day 2 onwards. Baseline clinical severity was compared between the two groups using chi-square tests to check whether these were similar in both groups at the start of the treatments.

Logistic regression was used to assess associations between baseline sign severity and survival outcome (alive or dead). Baseline signs that were statistically significant at the 5% level were included in the final logistic model and further analyzed using survival analysis. Death (either natural or via euthanasia) was considered as an event, whereas recovery was treated as censoring.

The association between sign severity at baseline and survival probability was assessed using the log-rank test. We also evaluated differences in survival between sheep that died naturally and those that were euthanized. Hazard ratios were calculated for signs that were significant in the survival model. Statistical significance was set at *P* < 0.05.

## Results

**Fig. 1:**
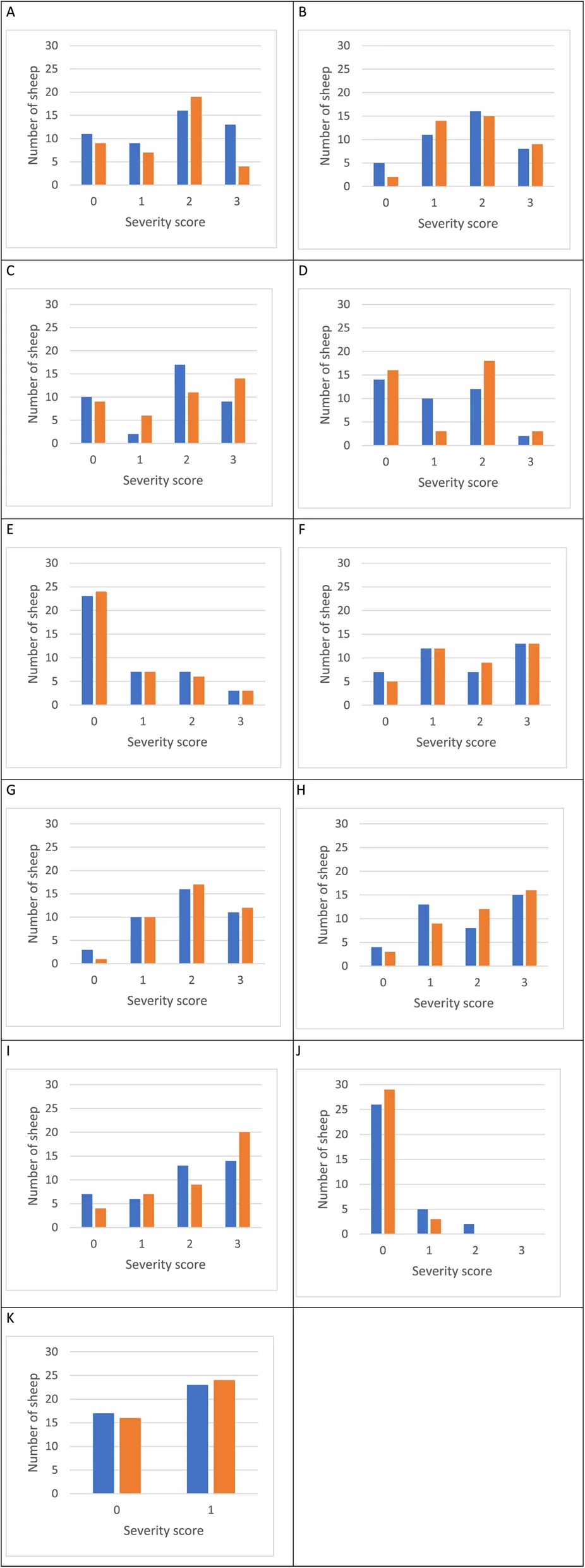
Number of sheep with highest scores of included clinical signs for all sheep in the two treatment groups during the study period: **A** Abnormal posture; **B** Swollen head; **C** Decreased feed intake; **D** Increased respiratory rate; **E** Lesions on the nose; **F** Lameness; **G** Nasal discharge; **H** Depression; **I** Excessive salivation; **J** Teat lesions; **K** Dyspnea. Observations of sheep that were not additionally treated with antihistamine (NAH) group are represented by the blue bars, whereas sheep that did also receive antihistamine (AH) are represented by the orange bars. Clinical signs were scored as 0 (not observed), 1 (mild), 2 (moderate), or 3 (severe). Only dyspnea was score as 0 (not observed) or 1 (observed)

A total of 83 sheep were clinically diagnosed with bluetongue during weekdays (Monday to Friday) and included in the study. An additional 38 sheep, diagnosed over the weekend, were excluded as it was logistically not possible to start the standardized treatment and monitor the sheep during those days. Highest scores of included clinical signs for all sheep during the study period are presented in Fig. 1. It appeared that, during the study period, many sheep had high scores for salivation, and low scores for lesions on the nose and on teats. Scores for abnormal posture, swollen head, decreased feed intake and increased respiratory rate were variable.

At baseline (day 0 or 1), the most frequently observed severe clinical signs in both groups were excessive salivation (28%), dyspnea (24%), depression (21%), and lameness (19%). No significant differences in the severity of baseline clinical signs were found between the antihistamine (AH; *n* = 42) and non-antihistamine (NAH; *n* = 41) groups (*p* > 0.05).

During the post-baseline period (day 2 and onward), the most frequently observed severe signs were dyspnea (62%), excessive salivation (44%), nasal discharge (33%), lameness (31%), and depression (29%).

The overall case fatality rate was 57%, with only 36 of 83 sheep surviving the infection. Among the 47 sheep that died, 34 (72.3%) were euthanized as they worsened in the opinion of the animal caretaker, whereas 13 (27.7%) died naturally. The AH group had a numerically higher case fatality rate than the NAH group, with a difference of 18.5%. However, the 95% confidence interval for this difference ranged from –0.01 to 0.38, indicating that it was not statistically significant.

Among all the baseline clinical signs, only the severity of salivation was significantly associated with survival outcome. Compared with those with a score of 0, those with a severity score of 3 had 18.6 times greater odds of dying (95% CI: 3.1–111.4). A trend was observed suggesting that higher depression scores might be associated with increased mortality, but this did not reach statistical significance (*p* = 0.08).

**Fig. 2:**
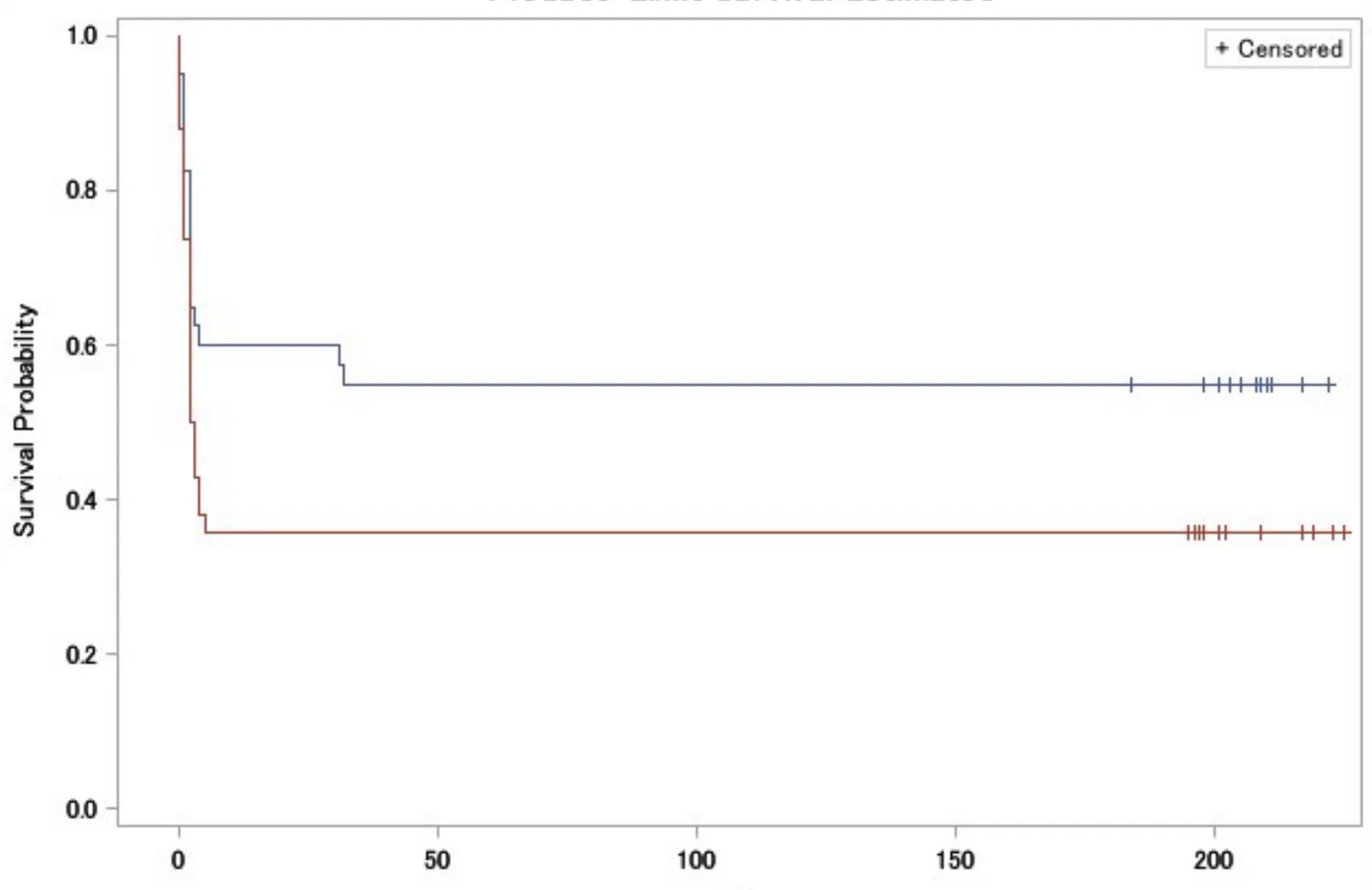
Survival of BTV-3 affected sheep in days after implementation of a treatment protocol with (brown line) and without (blue line) antihistamines. Death (either natural or via euthanasia) was considered as an event, whereas recovery was treated as censoring

The median survival time in the AH group was 1 day. The median survival time for the NAH group could not be calculated, as the majority of these animals survived beyond the observation period. Mean survival times were therefore used and were 0.8 days for the AH group and 2.5 days for the NAH group, respectively (Fig. 2). This difference in survival was statistically not significant.

Survival analysis also confirmed that salivation severity was a strong predictor of mortality. Sheep with a salivation score of 3 (highest) had an 8.0 times higher hazard of death than sheep with a salivation score of 0 (absent) (95% CI: 1.8–35.4).

## Discussion

Our findings support previous reports that BTV infections can result in high case fatality rates in naïve sheep, even when evidence based supportive treatment protocols are applied. Our protocol aimed to improve animal welfare and survival and included routine patient monitoring, NSAID administration, and oral paracetamol. Nevertheless, the result was poor in many cases.

It is important to note that our policy of euthanizing animals whose condition worsened, may have contributed to the observed fatality rate. However, given the severity of the observed clinical signs and the fact that many sheep died naturally, we believe that the impact of this policy on the results of the study was limited. Although we did not quantify this directly, both veterinary students and animal caretakers reported that the daily care and treatment regimen, especially the thrice-daily paracetamol administration, was labor intensive and emotionally demanding due to the severity of the clinical signs and the high case fatality rate. Nonetheless, they appreciated the structured protocol and its role in guiding timely decisions on whether to continue treatment or proceed with euthanasia.

As the Netherlands was officially free of BTV prior to September 2023, all sheep in our study were immunologically naïve. This likely contributed to the rapid spread of the virus and the severity of the observed clinical signs. The outbreak placed an unforeseen significant burden on animal care staff, necessitating extended work shifts and the involvement of many students. In this setting, quite different from a controlled trial, we opted for alternating treatment allocation to reduce operational complexity and the risk of misclassification, knowing that complete randomization would have been ideal. Although this approach has some methodological limitations, we believe that the uniformity of environmental and husbandry conditions across the flock likely minimizes the effects of confounding factors. However, we acknowledge that the approach made it impossible to study or correct for the effects of age and gender.

The first BTV-3 clinical cases on the farm were confirmed with PCR, while a large proportion of the neighboring cow herd also tested positive for BTV-3. All clinical observations and the propagation pattern of the infection within the sheep herd were clearly consistent with a BTV-3 infection and with reports from other affected sheep farms in the Netherlands [2]. As there were also no clinical indications for the circulation of other infections in the flock before the BTV-3 introduction, we are confident that all included cases were infected with BTV-3. Of course, we cannot exclude the possibility that the presence of other infections complicated the course of infection. In that case, we assume that complications occurred similarly in both groups as they were part of the same flock.

Antihistamines have immunomodulatory effects in Gram-negative bacterial infections, likely due to the ability of some bacteria to produce histamine and disrupt neutrophil responses [3]. There is, however, no evidence to support similar effects in viral infections such as BTV-3, although it is possible that the treatment may be effective in cases where secondary bacterial infections appear. We found no data on the effectiveness of on farm treatment protocols for bluetongue in sheep, including those involving antihistamines. Also, in a comparable hemorrhagic fever as Dengue in humans, the effects of antihistamine treatments did not improve the clinical endpoints [14]. In our study, the mean survival time was short in both treatment groups, and no significant improvement in survival was observed in the group receiving chlorphenamine. In fact, the AH group had a numerically shorter survival time, suggesting that this treatment may even be detrimental. We cannot exclude the possibility that these observations are due to the lack of power of our study. However, on the basis of these findings, we cannot recommend the use of antihistamines such as chlorphenamine in the treatment of bluetongue-affected sheep.

The case fatality rate of the on farm treatment for BTV-3 infected sheep in this study was clearly higher than observed in a study in hospitalized sheep with BTV infections [5]. Likely, this difference is likely partly due to the involved BTV serotype, as this largely determines disease severity. Another reason for the high case fatality rate in this trial is that we decided to euthanize patients for humanitarian reasons quite early, as we could not provide the intensive care that an animal hospital can provide.

In terms of clinical signs, severe lesions on the nose and on teats were not frequently observed during the study period, but dyspnea, excessive salivation, nasal discharge, lameness, and depression were commonly observed. Many sheep had also an abnormal posture, swollen head, decreased feed intake and increased respiratory rate during the study, but the severity was variable. This may reflect the variation in clinical presentation, but we acknowledge that it could also be due to difficulties with the correct and consistent scoring of the parameters by the students, despite their training.

Among the clinical signs, only the severity of salivation at baseline was significantly associated with survival, although the wide confidence interval around the odds ratio suggests caution in interpreting this result.

Recognizing that our findings may not apply to other BTV serotypes or epidemiological settings, we conclude that predicting survival in BTV-3-infected sheep on the basis solely of clinical signs is difficult. Furthermore, in naïve populations, survival can be very limited, even with intensive supportive care. Given the absence of evidence for a survival benefit from antihistamines, we recommend focusing on timely and humane decision-making regarding treatment continuation to minimize unnecessary animal suffering.

## Data Availability

The data that support the ﬁndings of this study are available from the corresponding author upon reasonable request.
